# NPM1 activates metabolic changes by inhibiting FBP1 while promoting the tumorigenicity of pancreatic cancer cells

**DOI:** 10.18632/oncotarget.4167

**Published:** 2015-06-05

**Authors:** Yi Zhu, Minmin Shi, Hao Chen, Jiangning Gu, Jiaqiang Zhang, Baiyong Shen, Xiaxing Deng, Junjie Xie, Xi Zhan, Chenghong Peng

**Affiliations:** ^1^ Department of Surgery, Ruijin Hospital, Shanghai Jiao Tong University School of Medicine, Shanghai, P.R. China; ^2^ Research Institute of Digestive Surgery, Ruijin Hospital, Shanghai Jiao Tong University School of Medicine, Shanghai, P.R. China

**Keywords:** pancreatic ductal adenocarcinoma, NPM1, FBP1, warburg effect

## Abstract

The nucleophosmin (*NPM1*) activates cancer development and progression in many malignant tumors. However, the regulatory role and underlying mechanisms of NPM1 in pancreatic cancer are unknown. In this study, we showed that NPM1 was up-regulated in PDAC, which indicated a poor prognosis. We also identified NPM1could stimulate aerobic glycolysis and repress fructose-1, 6-bisphosphatase 1 (*FBP1*) in pancreatic cancer cells. Restoring FBP1 expression partially reversed the tumor-promoting effects of NPM1, while the loss of FBP1 in PDAC tissues was indicative of a poorer prognosis. In sum, NPM1 promotes aerobic glycolysis and tumor progression in patients with pancreatic cancer by inhibiting FBP1.

## INTRODUCTION

*NPM1* (*nucleophosmin* or *B23*) is a multifaceted nucleolar protein that is involved in several cellular processes, including ribosome biogenesis [1], nucleocytoplasmic transport, centrosome duplication [2, 3], embryonic development [4], histone chaperone function, and transcriptional regulation [5, 6]. A frameshift mutation occurs in *NPM1* at its nucleolar localization motif, a redundant which will lead to nuclear-cytoplasm shuttling to result in development of a special type of AML [7, 8]. On the other hand, some groups have reported that *NPM1* gene mutations are typically absent in common solid cancers [9], which suggests that the *NPM1* mutation may not play a role in the tumorigenesis of common solid cancers.

Pancreatic ductal adenocarcinoma (PDAC) is one of the most lethal human malignancies. The average survival time after diagnosis with PDAC is usually less than 6 months, and the five-year survival rate is less than 3% [10]. The early detection of PDAC is uncommon, because it is typically asymptomatic until later stages (associated with poor prognosis). Therefore, at the time of diagnosis, as few as 10–15% of PDAC NPM1 patients are considered eligible for surgical resection [11]. For NPM1. A data from COSMIC (Catalogue of somatic mutations in cancer) database tested a total of 1242 pancreatic cancer specimens showed that none of them occurs point mutation. However, many new mechanisms have been identified that support the hypothesis that NPM1 can promote tumorigenesis in solid cancers via up-regulation. NPM1 can inhibit p53-mediated cellular senescence in adenomas and cancers of the colon [12]. NPM1 can promote cellular/tumor growth via novel NPM-BAX death evasion pathways in liver cancer [13]. Overall, high expression of NPM1 is common in rapidly and continuously proliferating cells and cancer cells. However, the relationship between NPM1 and pancreatic cancer remains unclear.

The Warburg effect is a shift from oxidative phosphorylation to glycolysis, a feature of which is increased lactate production even at normal oxygen conditions. As a result, it can promote cancer development and progression [14, 15]. Previous studies have reported that uptake of glucose increased in pancreatic cancer, so that ^18^F labeled fluorodeoxyglucose positron emission tomography is a good tool for diagnosis of and prognosis for pancreatic cancer [16, 17].

In this study, we demonstrated that NPM1 expression is up-regulated in pancreatic ductal adenocarcinomas, while elevated expression in tumor tissues may be linked to a poorer prognosis. Knock-downs of NPM1 in pancreatic cancer cell lines likely impair glucose uptake and lactate production. As a result, pancreatic cancer cell lines grow more slowly compared to control cells. We found that NPM1 inhibits Fructose-1,6-bisphosphatase 1 (FBP1) through transcriptional regulation. Furthermore, FBP1 appears to be a good prognostic biomarker for PDAC. Our findings shed light on the molecular mechanisms of how *NPM1-FBP1* axis controls tumor proliferation.

## RESULTS

### NPM1 is upregulated and associated with advanced disease in PDAC

**Figure 1:**
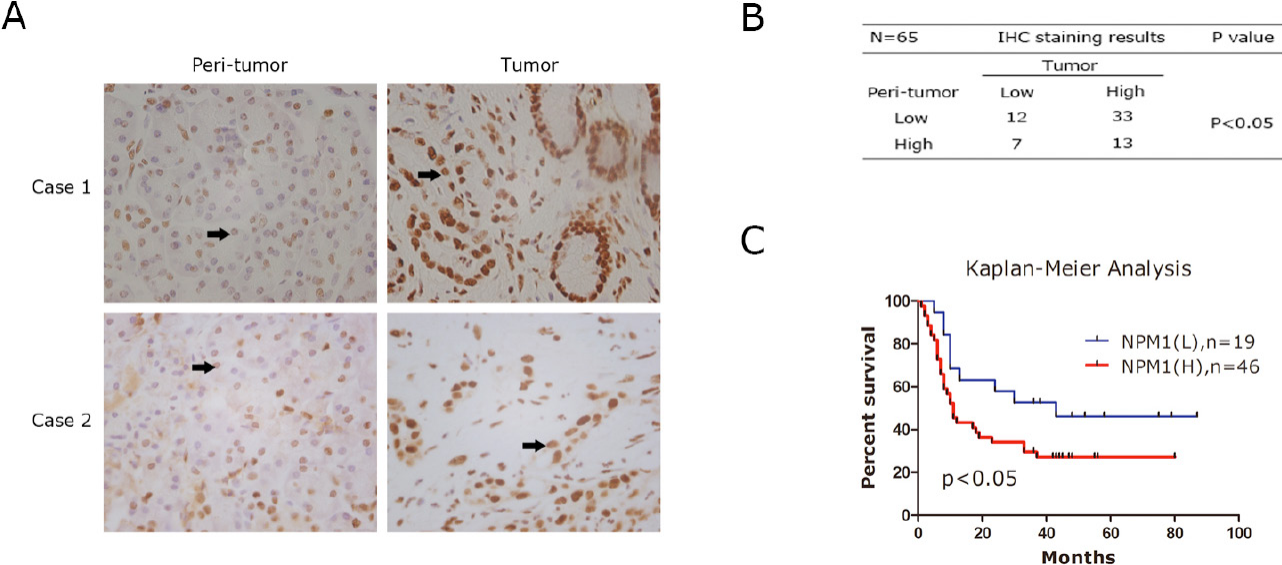
Immunohistochemistray results of NPM1 in PDAC specimens **A.** IHC staining to detect NPM1 in paired human PDAC specimens (tumor vs. peri-tumor). Arrows, positively stained cells. **B.** The Fisher's exact test of the IHC staining of 65 cases of the paired PDAC specimens on TMA chips. Each group was shown by the distribution of IHC staining scores for each case. Only IHC scores ≥ 4 was considered high. *n* = 65; *, statistical significance, *P* < 0.05. **C.** Survival analysis of PDAC patients by Kaplan-Meier plots and log-rank tests. Patients were categorized by high and low expression of NPM1 based on IHC staining scores. H, high; L, low.

A high-density tissue microarray was stained with an anti-human NPM1 antibody. Representative images indicated that expression of NPM1 in tumor tissues was higher than matched peri-tumor tissues, as shown in Figure 1A. Quantitative analysis confirmed that high staining scores of NPM1 also exist in peri-tumor tissues, but the proportion of high staining scores is significantly less than in tumor tissues (Figure 1B). To investigate the correlation between NPM1 and the prognosis of pancreatic cancer, we analyzed the IHC staining results combined with postoperative fellow-up data. We found that patients with relatively low NPM1 expression had a better prognosis compared to those with high NPM1 expression (*P* < 0.05; Figure 1C).

### NPM1 promotes progression of pancreatic cancer cells

**Figure 2:**
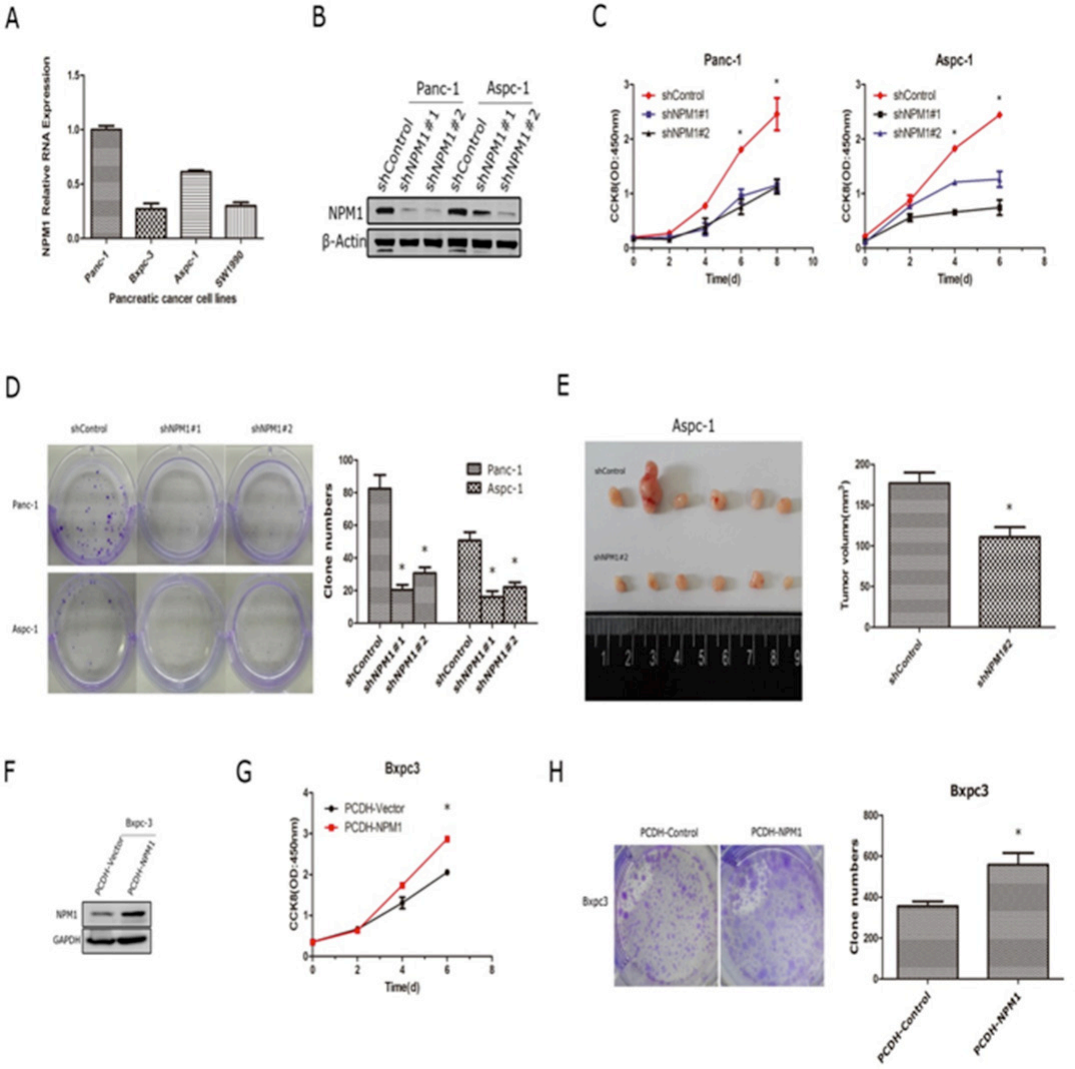
NPM1 promotes the progression of pancreatic cancer cells **A.** mRNA expression levels of NPM1 in four pancreatic cancer cell lines (Panc-1, Bxpc-3, Aspc-1, Sw1990). **B.** Identification of NPM1 stable knock-down in Panc-1 and Aspc-1 cell lines **C.** Cell counting assay (*n* = 3 technical replicates) in Panc-1 and Aspc-1 stable cell lines **D.** Colony forming assay (*n* = 3 technical replicates) and statistical results in Panc-1 and Aspc-1 stable cell lines **E.** Tumors removed from nude mice (*n* = 6 biological replicates) were measured, and the largest tumor in the control group has been excluded in statistical results **F.** Identification of NPM1 stable over-expression in the Bxpc3 cell line **G.** Cell counting assay in the Bxpc3 stable cell line (*n* = 3 technical replicates) **H.** Colony forming assay and statistical results in the Bxpc3 stable cell line (*, statistical significance, *P* < 0.05) (*n* = 3 technical replicates).

We had showed that the staining intensity of NPM1 correlates with the prognosis of pancreatic cancer. Therefore, we hypothesized that NPM1 can promote the progression of pancreatic cancer (via enhancing cancer cell growth). To study this possibility, we obtained four pancreatic cancer cell lines (Panc-1, Bxpc-3, Aspc-1, Sw1990) and tested the mRNA and protein levels of *NPM1* in each (Figure 2A and Supplementary Figure 1). We found that Panc-1 and Aspc-1 exhibited higher levels of NPM1 expression compared to it in the Bxpc-3 and sw1990. Therefore, we tried to knock-down NPM1 in Panc-1 and Aspc-1 and over-express it in Bxpc-3. We used lenti-virus packing two different short hairpins (shNPM1#1 and d shNPM1#2) to construct the stable cell lines by puromycin selection. First, we efficiently knock-down NPM1 in Panc-1 and Aspc-1(Figure 2B). Next, we found that knock-down of NPM1 dramatically attenuates cell proliferation (Figure 2C) and colony forming abilities (Figure 2D) in these two cell lines. We also injected two groups of Aspc-1 cells (Control and shNPM1#2) into nude mice to observe subcutaneous tumor formation. Knock-down of NPM1 in Aspc-1 cells slowed the speed of tumor growth *in vivo* (Figure 2E). In addition, we used a lentiviral vector to over-express NPM1 in the Bxpc-3 cell line (Figure 2F) and repeated cell proliferation and colony forming assays in this cell line (Figure 2G and 2H). We found that over-expression of NPM1 in Bxpc-3 cell line can accelerate the proliferation and enhance colony formation, which is consistent with our NPM1 knock-down results.

### NPM1 stimulates glucose uptake and lactate generation, while inhibiting FBP1 expression in pancreatic cancer cells

**Figure 3:**
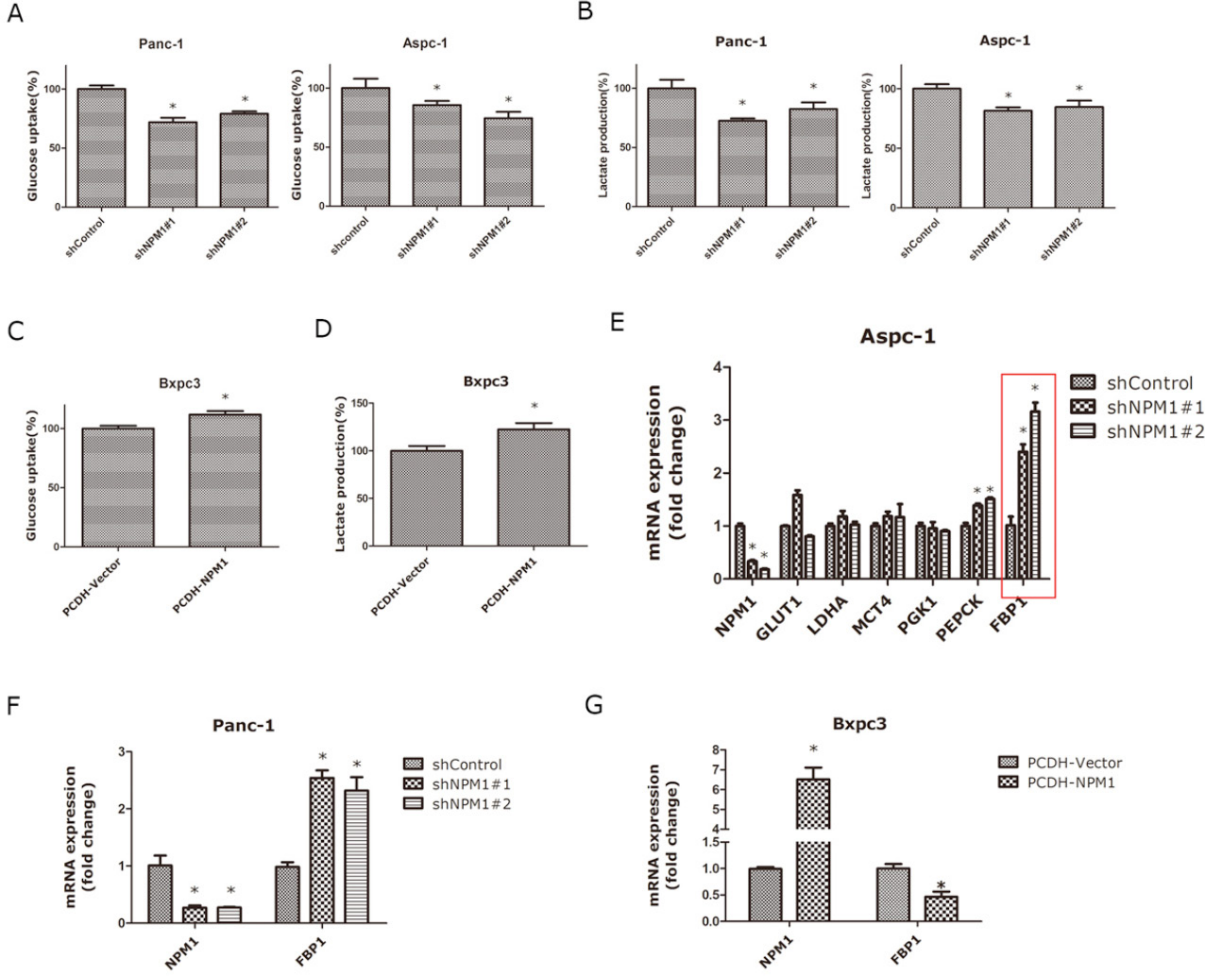
NPM1 regulates glucose uptake and lactate generation, while inhibiting FBP1 expression in pancreatic cancer cells **A.** Glucose uptake assay in Panc-1 and Aspc-1 stable cell lines **B.** Lactate production assay in Panc-1 and Aspc-1 stable cell lines **C.** Glucose uptake assay in Bxpc3 stable cell lines **D.** Lactate production assay in Bxpc3 stable cell lines **E.** mRNA expression of glucose metabolism-associated enzymes in the Aspc-1 stable cell line. **F.** mRNA expression of *FBP1* in Panc-1 and Bxpc3 stable cell lines **G.** mRNA expression of FBP1 in Bxpc3 stable cell lines (*, statistical significance, *P* < 0.05) (all the experiments above have three technical replicates).

To further examine whether NPM1 can affect the glucose metabolism in pancreatic cancer cells, we measured glucose uptake and found that knock-down of NPM1 significantly decreased glucose uptake in Panc-1 and Aspc-1 cell lines (Figure 3A), whereas over-expression of NPM1 enhanced glucose uptake in the Bxpc-3 cell line (Figure 3C). To examine whether NPM1 alters glucose metabolism via a switch from aerobic glycolysis to oxidative phosphorylation, we measured lactate production and found that the NPM1 over-expressing Bxpc-3 cell line produced more lactate than the vector control cells (Figure 3D), whereas NPM1-knockdown in Panc-1 and Aspc-1 cell lines resulted in less lactate production (Figure 3B). To identify the mechanism by which NPM1 enhances glucose metabolism, we selected several glucose metabolism associated enzymes (GLUT1, LDHA, MCT4, PGK1, PEPCK, FBP1) for further investigation. In the NPM1-knockdown Aspc-1 cell line, we found that PEPCK and FBP1 were up-regulated compared to the control groups in both two experiment groups (shNPM1#1 and d shNPM1#2), while FBP1 showed the a higher fold-change (Figure 3E). So we chose FBP1 for further investigation. To validate, we repeated the experiments in Panc-1(Figure 3F) and Bxpc3 cells (Figure 3G). These results support the results with the Aspc-1 cell line, which ultimately indicate that *FBP1* could be a newly identified target gene of NPM1.

### FBP1 is a direct target gene of NPM1

**Figure 4:**
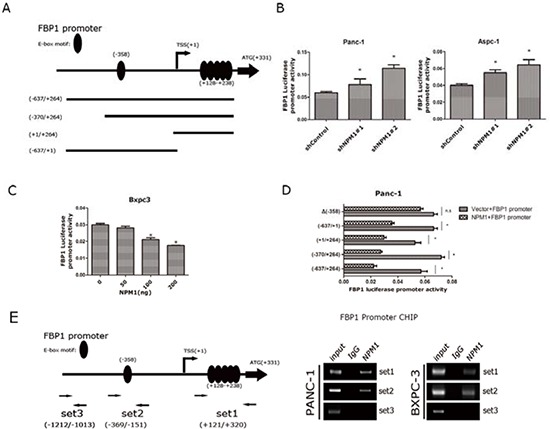
*FBP1* is a direct target of NPM1 **A.** Schematic map of *FBP1* promoter and its truncation design **B.** Luciferase reporter assay in Panc-1, and Aspc-1 cell lines **C.** Luciferase reporter assay of *FBP1* promoter in Bxpc3 with different NPM1 doses **D.** Luciferase reporter assay of *FBP1* promoter and its truncations. Δ(−358) means E-box deletion in truncation (−637/+1) **E.** The map of CHIP primers and RT-PCR results of CHIP. (*, statistical significance, *P* < 0.05) (all the experiments above have three technical replicates)

Next, we wanted to determine whether *FBP1* was in fact a direct target of NPM1. A previous study [20] had found that E-box motifs were concentrated in the promoter of *FBP1* (one in −358 before TSS and another five between +128 to +238 after TSS). E-box motifs have been identified as a major repressive motif, so we speculated that an E-box motif might be the NPM1 binding site in the *FBP1* promoter. An *FBP1* promoter luciferase reporter plasmid and its three truncations were constructed, as shown in Figure 4A. First, we transfected equivalent amounts of *FBP1* promoter plasmids into the NPM1-knock-down Panc-1 and Aspc-1 cell lines. In NPM1 knock-down cells, the luciferase activity of the *FBP1* promoter was significantly elevated compared to the control groups (Figure 4B). We transfected equivalent amounts of *FBP1* promoter plasmids into the Bxpc3 cell line as well and added a concentration gradient of NPM1 plasmids. As the concentration of NPM1 increased, the luciferase activity of the *FBP1* promoter decreased (Figure 4C). The NPM1 plasmid was also transfected with *FBP1* promoter truncations and mutations, and repression was attributed to the existence of E-box motifs. However, when the E-box motif was deleted at −358 in truncation −638/+1, the repression was no longer obvious (Figure 4D), which suggests that this E-box motif could be a potential binding site for NPM1. To examine whether NPM1 binds to the FBP1 promoter, we performed chromatin immunoprecipitation (ChIP) in Panc-1 and Bxpc3 cell lines using three sets of primers (Figure 4D). ChIP showed that NPM1 does bind to the promoter of *FBP1* at the E-box. Collectively, these results indicate that *FBP1* is a direct target of NPM1.

### Restoration of FBP1 can partially reverse the effects of NPM1 on pancreatic cancer cells

**Figure 5:**
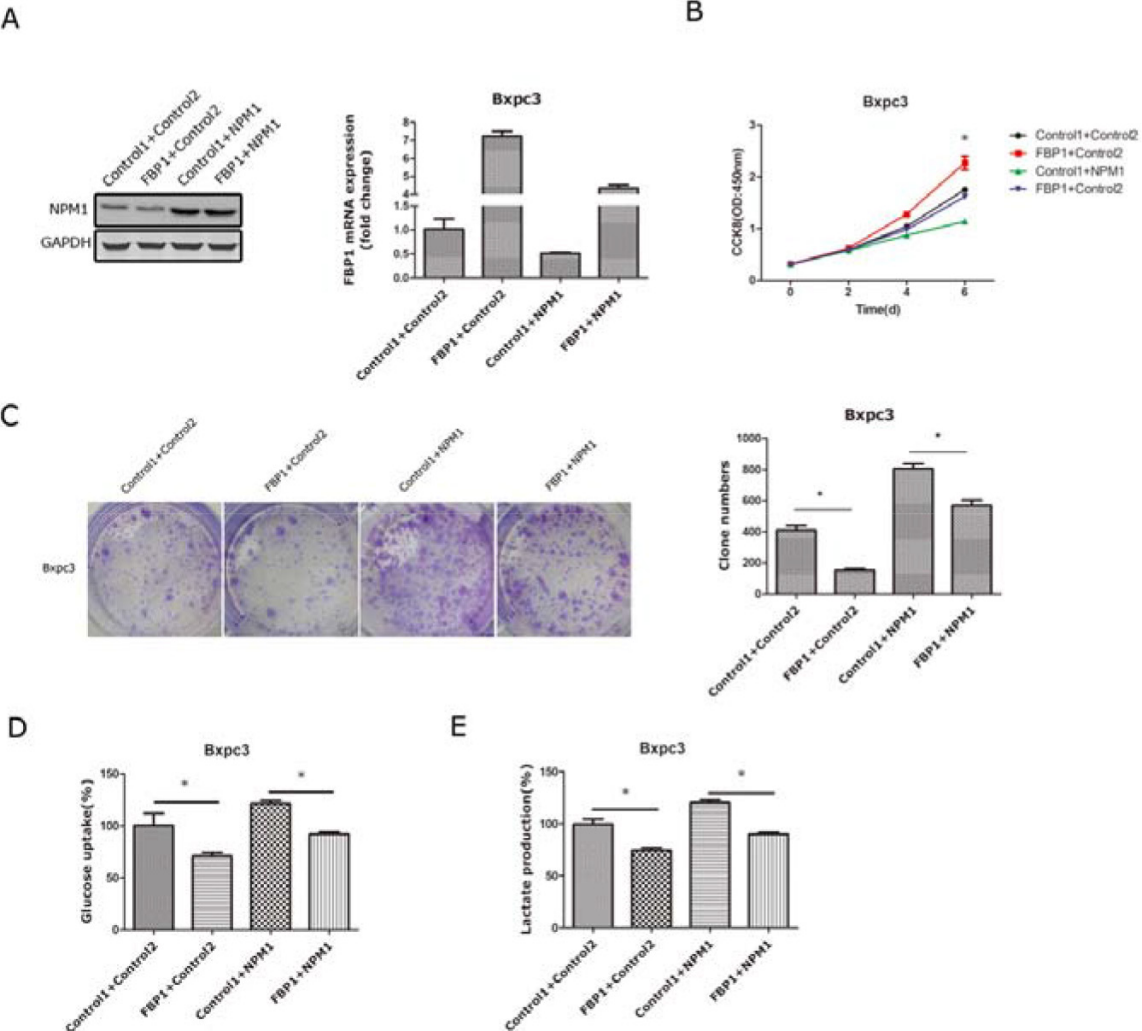
Restoration of FBP1 expression in pancreatic cancer cells with NPM1 overexpression **A.** Identification of NPM1 and FBP1 stable overexpression in the Bxpc-3 cell line(Control means PCDH vector) **B.** Cell counting assay in the Bxpc3 stable cell line **C.** Colony forming assay and statistical results in the Bxpc-3 stable cell line **D.** Glucose uptake assay in the Bxpc3 stable cell line **E.** Lactate production assay in the Bxpc3 stable cell line (*, statistical significance, *P* < 0.05) (all the experiments above have three technical replicates).

To further demonstrate that FBP1 is a critical target gene involved in the NPM1-induced phenotypes of pancreatic cancer cells, we over-expressed FBP1 and NPM1 in the Bxpc-3 cell lines, and effective restoration of FBP1 expression in Bxpc3 cells was confirmed by qRT-PCR. Exogenous FBP1 (Figure 5A) significantly reduced proliferation and colony forming abilities in Bxpc3 (FBP1+Control2) cells compared with the Bxpc3 (Control1+Control2). However, in Bxpc3 (NPM1-overexpressing) cells, FBP1 partially reversed the increased growth and colony forming abilities caused by NPM1 over-expression (Figure 5B and 5C). Glucose uptake and lactate generation assays were also tested in these four cell lines. As expected, exogenous FBP1 significantly reduced glucose uptake and lactate generation in Bxpc3 (Control2 group) cells. In Bxpc3 (NPM1-overexpressing) cells, FBP1 could totally reverse glucose uptake and lactate generation (Figure 5D and 5E).

### FBP1 expression is inversely correlated with NPM1 in pancreatic cancer

**Figure 6:**
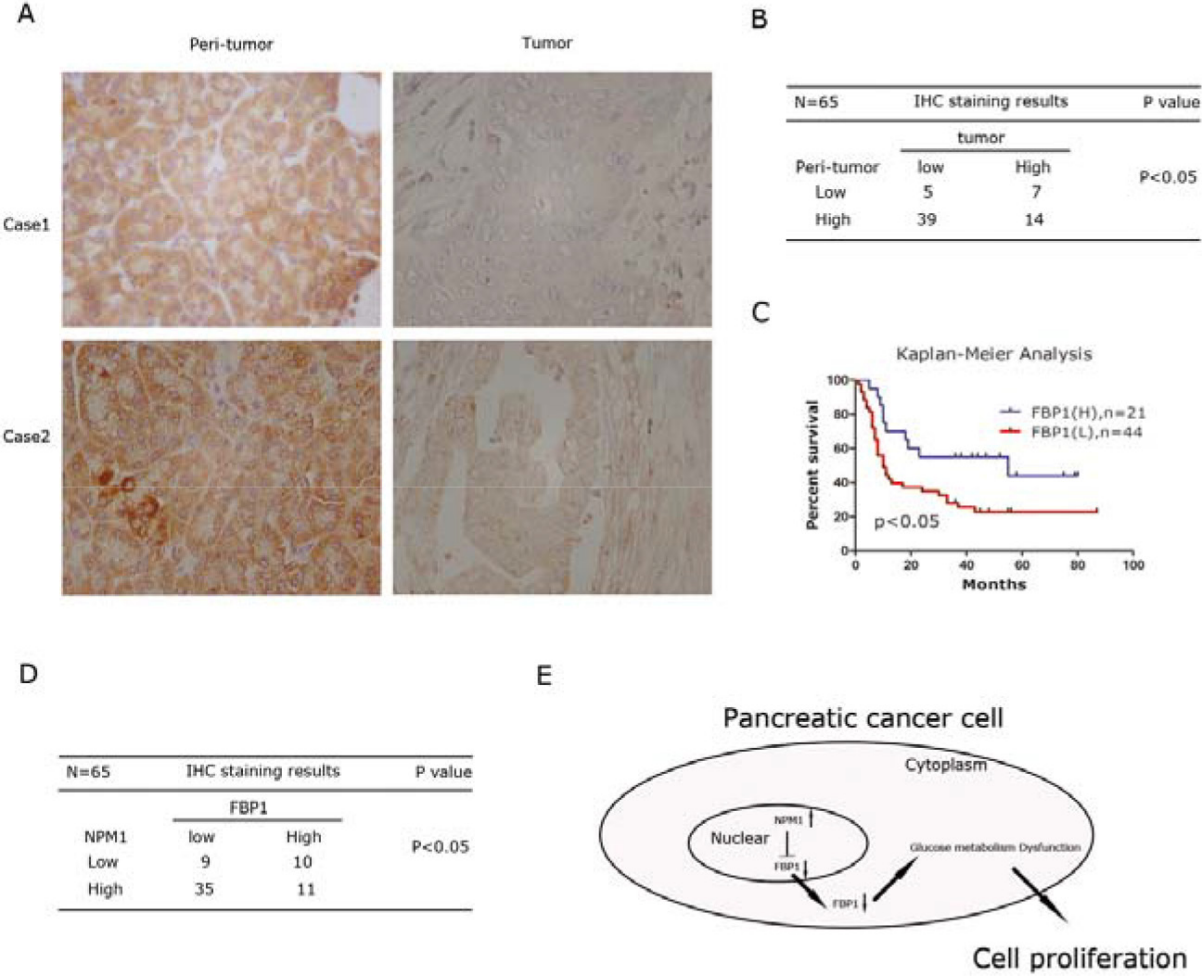
FBP1 expression is inversely correlated with NPM1 in pancreatic cancer **A.** IHC staining to detect FBP1 in paired human PDAC specimens (tumor vs. peri-tumor). **B.** The Fisher's exact test of the IHC staining of 65 cases of the paired PDAC specimens on TMA chips. Each group was shown by the distribution of IHC staining scores for each case. Only IHC scores ≥ 4 was considered high. *n* = 65; *, statistical significance, *P* < 0.05. **C.** Survival analysis of PDAC patients by Kaplan-Meier plots and log-rank tests. Patients were categorized by high and low expression of the FBP1 based on IHC staining scores. H, high; L, low. **D.** The statistical correlation between NPM1 and FBP1 expression in tumor tissues based on IHC staining scores. **E.** Model for *NPM1-FBP1* axis. NPM1 represses FBP1 via transcriptional regulation. Down-regulation of FBP1 will cause glucose metabolism dysfunction, which will lead to proliferation of pancreatic cancer cells.

Based on the above findings, we assessed whether FBP1 expression was associated with PDAC outcomes. Therefore, the tissue microarray was also stained with an anti-human FBP1 antibody. Representative images indicated that expression of FBP1 in tumor tissues was much lower than matched peri-tumor tissues, as shown in Figure 6A. Statistical analysis indicated that FBP1 was expressed at low levels in the majority of PDAC tissues. On the contrary, normal pancreas tissues always showed a high level of FBP1 expression (*P* < 0.05) (Figure 6B). Low levels of FBP1 expression also indicated a poorer PDAC prognosis (*P* < 0.05; Figure 6C). In addition, FBP1 expression was inversely correlated with NPM1 expression (*P* < 0.05; Figure 6D). We also delineated a small heatmap to illustrate this result (Supplementary Figure 2).

## DISSCUSION

In the present study, we determined the roles of NPM1 in pancreatic ductal adenocarcinoma (PDAC) aerobic glycolysis and of NPM1-FBP1 axis in pancreatic tumorigenesis. The results showed a critical role for NPM1 in regulation of aerobic glycolysis by the transcriptionally repressed *FBP1* gene. First, we found that NPM1 was up-regulated in pancreatic tumor specimens and associated with the prognosis. Second, reduced expression of NPM1 increased the expression of *FBP1* and impaired glucose uptake and lactate production, whereas over-expression of NPM1 did the opposite. These data indicated that NPM1 regulated aerobic glycolysis by inhibiting the expression of FBP1. Third, NPM1 bound directly to the *FBP1* promoter region at the E-box motif and regulated expression of *FBP1* at the transcriptional level. Fourth, restore the FBP1 in pancreatic cancer cells could reverse the NPM1-induced glucose metabolism dysfunction. At last, FBP1 was down-regulated in pancreatic tumor specimens and also associated with the prognosis of (PDAC). Thus, this novel *NPM1-FBP1* signaling critically contributed to the Warburg effect in pancreatic cancer cells and as a result, to pancreatic cancer development and progression.

NPM1 has been previously reported to act as an oncogene in many diseases, with the exception of PDAC [12, 13]. Studies of the mechanisms by which NPM1 promotes tumor growth mainly focus on ribosome biogenesis or its anti-apoptotic effects in the cytoplasm [13]. NPM1 can be acetylated at K residues and shuttle to the nucleoplasm from the nucleolus to participate in transcriptional regulation, the main function of NPM1 being transcriptional activation [21]. In this study, we identified NPM1 can also participate in transcriptional repression.

FBP1 was originally identified as a rate-limiting enzyme in gluconeogenesis. Recently, it was reported that FBP1 is often significantly down-regulated and acts as a tumot surpressor gene in basal-like breast cancers [22] and renal cancers [23, 24]. FBP1 down-regulation has been associated with the Warburg effect, which is a common metabolic characteristic of malignant tumors. Our results showed that FBP1 was also dramatically down-regulated in PDAC and at the same time was associated with a poor PDAC prognosis. Previous studies have reported that DNA hypermethylation of the *FBP1* promoter is chief cause of its repression in liver, colon, and gastric cancers [25, 26]. Therefore, the relationship between NPM1 and hypermethylation of *FBP1* promoter warrants further investigation.

In this study we found a novel role for NPM1 in metabolic regulation in pancreatic cancer. However, the specimens we used were all from patients with a resectable pancreatic cancer, and we also didn't know if the high expression of NPM1 was a final result of therapy. It was a limitation of our work and needed to be investigated in the future.

## MATERIALS AND METHODS

### Tissues microarray and immunohistochemistry (IHC)

Tissue microarray (TMA) chips that contain 80 cases of paired PDAC tumor and peri-tumor specimens were purchased (HPan-Ade180 Sur-01; Shanghai Outdo Biotech Company). All specimens spotted on TMA chips included complete postoperative follow-up for a period of 3 to 7 years. Tumor staging was evaluated according to the TNM classification of malignant tumors. Before IHC staining, all specimens were evaluated by hematoxylin-eosin staining to ensure the pathological types. Only those with a pathological diagnosis of PDAC were considered as tumor samples. A total of 65 cases were qualified for further immunohistochemistry investigation (Supplementary Table 1). The following primary antibodies were used: anti-NPM1 (ab52664), and anti-FBP1 (ab109020). All antibodies above were obtained from Abcam and diluted in 0.1 M phosphate buffered saline (PBS). After deparaffinization and rehydration, antigen retrieval was performed by immersing the slides in antigen retrieval buffer [10 mM sodium citrate, pH 6.0) at 95°C for 30 min then treating with Tris-buffered saline containing 0.1% Triton X-100 (TBST, pH 7.6) for 5 min. Endogenous peroxidases were blocked with 3% hydrogen peroxide for 20 min. After washing three times with 0.01 M PBS, nonspecific binding was blocked with goat serum for 30 min. Tissues were then incubated with primary antibodies at 4°C overnight followed by incubation with a peroxidase-labeled polymer conjugated to anti-rabbit immunoglobulin (Invitrogen) for 1 h at 37°C. After washing with 0.01 M PBS, diaminobenzidine (Boster) was used to visualize tissue antigens for 2 minutes. The sections were counterstained with hematoxylin and dehydrated. All slides were examined and scored by two of the pathologists who were blinded to clinical patient data. The IHC score is calculated by combining the quantity score (percentage of positive stained cells) with the staining intensity score. The quantity score ranges from 0 to 4, i.e. 0, no immunostaining; 1, 1–10% of cells are stained; 2, 11–50% are positive; 3, 51–80% are positive; and 4, ≥ 81% of cells are positive. The staining intensity was scored as: 0 (negative), 1 (weak), 2 (moderate) and 3 (strong). Raw data were converted to IHC score by multiplying the quantity score (0–4) by the staining intensity score (0–3). Theoretically, the scores can range from 0 to 12. An IHC score of 9–12 was considered a strong immunoreactivity; 5–8, moderate; 1–4, weak; and 0, negative [18, 19]. Samples whose IHC score were more than 4 were considered to be high, and less than 4 were considered to be low.

### Plasmids

*NPM1* and *FBP1* full-length cDNA were cloned from Panc-1 cDNA and then inserted into the Lenti-virus vector PCDH between the *Hind* III and *Eco*RI restriction sites. Two shRNAs (purchased from Sigma-Alrich) targeting *NPM1* were inserted in lenti-virus vector pLKO.1. The sequences targeting *NPM1* were: shNPM1#1: 5-gcgccagtgaagaaatctata-3 and shNPM1#2: 5-cctagttctgtagaagacatt-3 and the shControl was the vector pLKO.1 as a negative control. *FBP1* promoter plasmid (−637/+264) was cloned from genomic DNA and inserted to the PGL3-vector. Three *FBP1* promoter truncations (−370/+264,+1/+264,−638/+1) were constructed from the *FBP1* promoter plasmid (−637/+264), as described above. An *FBP1* promoter mutation whose E-box motif at −358 were deleted was constructed followed from *FBP1* promoter truncation (−638/+1). *NPM1* cDNAs were also inserted to the pcDNA 3.1-Flag vector.

### Cell culture

HEK-293T and pancreatic cancer cells Panc-1, Aspc-1, and Bxpc-3 were obtained from the ATCC and were tested and authenticated by DNA typing at the Shanghai Jiao Tong University Analysis Core. The cells were maintained in DMEM or RPMI 1640 (Gibco) supplemented with 10% FBS (Gibco), 2 mmol/l L-glutamine (Hyclone), and penicillin [50 U/ml) / streptomycin [50 μg/ml) (Hyclone) at 37°C under 5% CO_2_ in a humidified chamber.

### Virus packing and infection

Lenti-virus plasmids mentioned above was transfected into HEK-293T with virus packing plasmids to produce the lenti-virus. The reagent of transfection was Lipofectamine2000 (Invitrogen). The viral supernatants were collected at 48 and 72 hours after transfection and ten added, into Panc-1, Aspc-1, and Bxpc-3 cells to construct the stable transfected cell lines. Puromycin was added into the media after 48 hours of infection and maintained for at least 1 week to select stable transfected cell lines.

### Cell growth counting and clone formation assays

For cell growth counting assays, multiple cultures of pancreatic cancer cells were plated in 96-well plates at a density of 1.5 × 10^3^ cells per well. Every two days one set of cultures was tested followed by cell counting kit-8 at 450 nm. For clone formation assays, pancreatic cancer cells were seeded into 6-well plates at a density of 1.5 × 10^3^ cells per well. After 2 or 3 weeks of growth, the cells were washed in PBS 3 times, fixed in methanol, and dyed with methylrosanilnium chloride solution at a 0.01% concentration for counting.

### Glucose uptake and lactate production assay

For Glucose uptake assay, a total of 1 × 10^3^ pancreatic tumor cells was prepared in 96-well plates using a Colorimetric glucose uptake assay kit (BioVision). For lactate production assay, a total of 1 × 10^6^ pancreatic tumor cells was prepared in 10 cm dishes were measured by lactate production assay kits from BioVision.

### Western blot

Standard Western blotting, which was previously described [20], was carried out using whole cell protein lysates and a primary antibody against NPM1 (abcam:ab52664) and a secondary antibody (anti-rabbit IgG, Santa Cruz Biotechnology). Equal protein specimen loading was monitored using an anti-GAPDH antibody (cell signaling technology) or anti-β-actin antibody (Cell Signaling Technology).

### Quantitative RT-PCR

Total RNA was isolated from cells with TRIzol reagent (Invitrogen). Next, 2 μg of total RNA was reverse transcribed using a First Strand cDNA kit (Invitrogen) to synthesize cDNA template, qRT-PCR was performed on the 7500 Fast Real time PCR system (Applied Biosystem) using SYBR Green agent (Applied Biosystem). Primers used for qRT-PCR assay are listed in the Supplementary Table 2. All PCR assays were repeated three times.

### Chromatin immunoprecipitation

The chromatin immunoprecipitation (ChIP) experiments were performed in Panc-1 and Bxpc3 cells. To prepare cells for ChIP assays, the Panc-1and Bxpc3 cells were grown in 10 cm plates to 90% confluency for use with a CHIP kit (Thermo scientific). The immunoprecipitated DNA fragments were detected by RT-PCR assays. The primer sets that amplify the DNA fragment in the FBP1 promoter are as follows: Set1: forward, 5-ggcctggcccagttgcac-3; reverse, 5-gtagagcgcggggctgca-3. Set2: forward, 5-gacagaagggcc aggtga-3; reverse, 5-gccagagagaaagctatgactg-3. Set3: forward, 5-ttagctcctaattgtcagtcctg-3; reverse, 5-ggaggtgg tggattctggac-3 [20]. The PCR products were resolved electrophoretically on a 2% agarose gel and visualized using ethidium bromide staining.

### Luciferase reporter assay

For transfection, Panc-1, Aspc-1, and Bxpc-3 cells were seeded at 5 × 10^4^ cells per well in 24-well plates. The β-galactosidase plasmid (50 ng) and pGL3-FBP1- Luc reporter (200 ng), along with a pcDNA3.1-Flag-NPM1 plasmid, were transiently transfected into the cells. Twenty-four hours post-transfection, cells were harvested and lysed. The luciferase and β-galactosidase activities were measured with a luciferase reporter gene assay kit (Invitrogen) and a β-galactosidase kit (Invitrogen), respectively. The transfection efficiency across plates was normalized to β-galactosidase activity, and all transfections were repeated three times in duplicate.

### Subcutaneous tumor growth

All experimental animal procedures were performed in compliance of the institutional ethical requirements and were approved by the Shanghai Jiao-Tong University School of Medicine Committee for the Use and Care of Animals. To evaluate tumor growth *in vivo*, 2 × 10^6^ Aspc-1 (Negtive control and shNPM1#2) cells were prepared by cell counting and injected to the left scapular region of nude mice (Male, 6-week old, purchased from Slaccas Laboratory Animal). Tumor volumes were monitored every day, Animals were sacrificed 2 weeks after injection, and the tumors were removed completely for volume Calculation.

### Statistical analysis

Data are shown as the mean ± one standard deviation (SD) and data were analyzed by a Student *t* test. The distribution of the IHC scoring results for each protein on TMA chips was analyzed by the Fisher's exact test. The postoperative survival of patients with PDAC was analyzed by the Kaplan-Meier estimator and tested by the log-rank. *P* values < 0.05 were considered statistically significant. Statistical analysis was performed using IBM SPSS Statistics Version 20 software.

### Study approval

The study was approved by the Ethic and Research Committees of Ruijing Hospital, Shanghai Jiaotong University School of Medicine and was conducted in accordance with the Declaration of Helsinki Principles. The procedures for pancreatic tumor resection were described in detail to all patients before admission, and informed consent was obtained for all participating patients.

## SUPPLEMENTARY FIGURES AND TABLES




