# Supplementary material for: NPM1 activates metabolic changes by inhibiting FBP1 while promoting the tumorigenicity of pancreatic cancer cells
Source: Oncotarget. 2015 Jun 5;6(25):21443–51. doi: 10.18632/oncotarget.4167 (PMC4673277; doi:10.18632/oncotarget.4167)
Supplement: Supplementary file 1 [file oncotarget-06-21443-s001.pdf]

SUPPLEMENTARY FIGURES AND TABLES

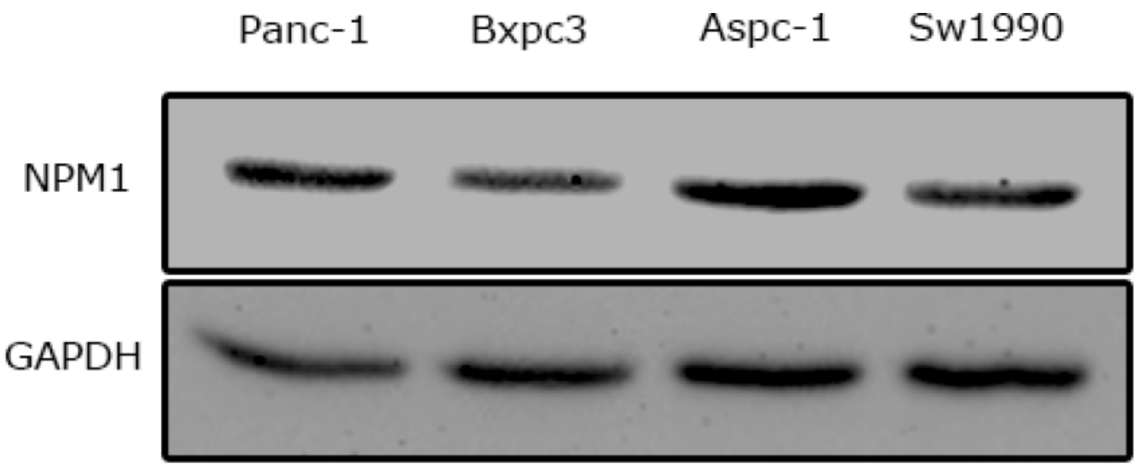

Supplementary Figure 1: The protein expression of NPM1 in four pancreatic cancer cell lines (Panc-1, Bxpc-3, Aspc-1 and sw1990).

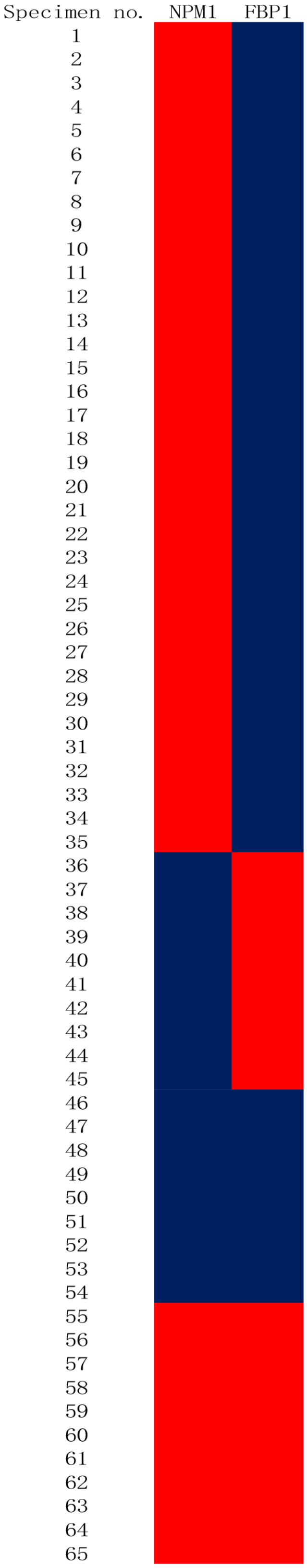

Supplementary Figure 2: The small heatmap for NPM1/FBP1 IHC results (Red stands for high and blue stands for low for both two proteins, *n* = 65).

Supplementary Table 1: The clinical and pathological features of 65 pancreatic cancer patients

Supplementary Table 2: The Quantitative RT-PCR primers mentioned in the article

primer	sequence
GLUT1-F	GATTGGCTCCTTCTCTGTGG
GLUT1-R	TCAAAGGACTTGCCCAGTTT
NPM1-F	GGGCTGGTGCAAAGGATGAG
NPM1-R	CAAGGGAAACCGTTGGCTGT
PGK1-F	CATACCTGCTGGCTGGATGG
PGK1-R	CCCACAGGACCATTCCACAC
FBP1-F	CTCTATGGCATTGCTGGTTCT
FBP1-R	TCCACTATGATGGCGTGTTTAT
LDHA-F	AAGCGGTTGCAATCTGGATTCAG
LDHA-R	GGTGAACTCCCAGCCTTTCC
MCT4-F	CAGTTCGAGGTGCTCATGG
MCF4-R	ATGTAGACGTGGGTCGCATC
PEPCK-F	CCCTGCGAGTGCTTAGTGG
PEPCK-R	GAAGGAGTTACAATCACCGTCT
